# Follow-up study of infants recruited to the randomised, placebo-controlled trial of azithromycin for the prevention of chronic lung disease of prematurity in preterm infants—study protocol for the AZTEC-FU study

**DOI:** 10.1186/s13063-022-06730-x

**Published:** 2022-09-21

**Authors:** Sarah J. Kotecha, Christopher W. Course, Kathryn E. Jones, W. John Watkins, Janet Berrington, David Gillespie, Sailesh Kotecha

**Affiliations:** 1 Department of Child Health, School of Medicine, Cardiff University, Heath Park, Cardiff, CF14 4XN UK; 2 Neonatal Intensive Care Unit, Newcastle Upon Tyne Hospitals NHS Foundation Trust, Newcastle Upon Tyne, UK; 3 Centre for Trials Research, College of Biomedical and Life Sciences, Cardiff University, Cardiff, UK

**Keywords:** Neonates, Preterm, Azithromycin, Neurodevelopment, Chronic lung disease, Bronchopulmonary dysplasia, Respiratory

## Abstract

**Background:**

Preterm birth, especially at less than 30 weeks’ gestation, is significantly associated with respiratory, neurodevelopmental and growth abnormalities. The AZTEC study has recruited 799 infants born at < 30 weeks’ gestation to determine if a ten-day intravenous treatment with azithromycin improves survival without development of chronic lung disease of prematurity (CLD) at 36 weeks’ post menstrual age (PMA) when compared to placebo. The follow-up studies will compare respiratory, neurodevelopmental and growth outcomes up to 2 years of corrected age between infants who received azithromycin and those who received placebo in the early neonatal period.

**Methods:**

Survivors at 36 weeks’ PMA from the main Azithromycin Therapy for Chronic Lung Disease of Prematurity (AZTEC) study with parental consent will continue to be followed up to discharge from the neonatal unit and to 2 years of corrected age. Length of stay, rates of home oxygen, length of supplemental oxygen requirement, hospital admissions, drug usage, respiratory illness, neurodevelopmental disability and death rates will be reported. Data is being collected via parentally completed respiratory and neurodevelopmental questionnaires at 1 and 2 years of corrected age respectively. Additional information is being obtained from various sources including hospital discharge and clinical letters from general practitioners and hospitals as well as from national databases including the National Neonatal Research Database and NHS Digital.

**Discussion:**

The AZTEC-FU study will assess mortality and important neonatal morbidities including respiratory, neurodevelopmental and growth outcomes. Important safety data will also be collected, including the incidence of potential consequences of early macrolide use, primarily pyloric stenosis. This study may have implications on future neonatal care.

**Trial registration:**

The study was retrospectively registered on ISRCTN (ISRCTN47442783).

## Background

Survival from preterm birth, especially at extremes of gestation, has improved significantly over the last 20 years [1]. However, survivors of preterm birth remain at risk of significant longer-term morbidity including for neurodevelopmental, respiratory and growth outcomes. Chronic lung disease of prematurity (CLD), also known as bronchopulmonary dysplasia, results from multiple exposures resulting in pulmonary inflammatory injury to the structurally immature lungs with subsequent altered lung growth which can adversely affect longer term lung function [2]. Despite advances in neonatal intensive care, CLD remains a significant medical concern, and rates have not improved significantly, possibly due to improved survival of more extremely immature infants (< 26 weeks’ gestation) [3]. Despite CLD being a diagnosis of the neonatal period, it is associated with longer term morbidity including increased hospitalisation in infancy [4, 5], increased wheezing and other respiratory symptoms [6] and decreased lung function [6–9]. The reduction in lung function is increasingly thought to have significant health implications for the individual’s lifespan, with premature development of chronic obstructive pulmonary disease (COPD) now considered a potential outcome of developing CLD in infancy [10].

Neurodevelopmental delay is another significant adverse outcome in survivors of preterm birth, with increased rates of cerebral palsy and moderate to severe developmental disability frequently noted at 2-year corrected gestational age [11, 12]. Risk factors for the development of CLD, such as low gestation, prolonged duration of respiratory support following birth, are also associated with poorer neurodevelopmental outcomes [13], and there is a strong association between CLD and longer-term adverse motor and cognitive function [14] and reduced IQ [15].

An additional adverse outcome after preterm birth is poor somatic growth. Postnatal growth is often compromised for preterm born infants, due to an immature gastrointestinal system, prolonged periods of parenteral nutrition, increased energy requirements especially during periods of illness, periods of fluid restriction and feed intolerance or due to gastrointestinal disorders such as necrotising enterocolitis. Preterm-born infants often continue to have reduced growth velocities leading up to discharge. Following discharge, infants, especially those who had CLD, are at high risk of poorer growth attainment [16]. Reduced head growth has been shown to have a link with later neurodevelopmental conditions such as cerebral palsy [17].

There have been many attempts to improve these outcomes. Azithromycin is a commonly prescribed antibiotic in the paediatric and adult populations for respiratory conditions associated with infection and inflammation such as cystic fibrosis [18] and COPD [19] and has been shown to improve the long-term outcome for these patients. In the paediatric population, use of macrolides such as erythromycin and azithromycin, may be associated with a small risk of complications such as cardiac rhythm abnormalities and pyloric stenosis [20], but it is unclear if these risks extend to the preterm neonatal population, and a recent systematic review did not find any such evidence [21]. The main azithromycin therapy for chronic lung disease of prematurity (AZTEC) trial has monitored these potential complications.

Azithromycin is attractive as it is active against *Ureaplasma* spp. which has been implicated in the development of CLD [22] and has potent anti-inflammatory effects which may target the pulmonary inflammation that is often observed at 7–10 days of age in preterm-born infants who develop CLD [23, 24]. Interestingly, azithromycin use has also shown improved neurodevelopmental outcomes in animal models of neuro-injury. In one study, 30 mg/kg azithromycin administered to neonatal rat model with right carotid ligation and 8% oxygen hypoxic exposure resulted in less functional defects, less weakness in left paw grip and less right hemisphere damage, in the treated group when compared to the placebo group [25]. In another study, azithromycin (20 mg/kg for 2 days) resulted in physiological (decreased paresis and co-ordination deficits) and histological (decreased ventriculomegaly and neuronal necrosis/apoptosis) improvements in a neonatal mouse model of periventricular leukomalacia [26].

The AZTEC study is an adequately powered phase III randomised, placebo-controlled trial examining whether a 10-day course of early azithromycin improves rates of survival without CLD in infants born at < 30 weeks’ gestation [27]. The primary outcome is survival without development of CLD when the active and placebo arms are compared. Secondary outcomes include rates of CLD and severity at 36 weeks’ PMA, mortality by 36 weeks’ PMA, number of days of respiratory support/oxygen-dependency, development of complications of prematurity, serious adverse events/reactions and resistance to macrolides in lung and stool samples [27]. It will also investigate the extent to which the outcomes differ dependent on whether the participants’ lungs were or were not colonised with baseline *Ureaplasma* spp.

It will be important to follow these infants beyond 36 weeks’ PMA to monitor any longer-term effects from the early use of azithromycin and ensure longer term safety. The AZTEC follow-up studies (AZTEC-FU) are assessing death rates, respiratory, neurodevelopmental and growth outcomes for AZTEC study participants from 36 weeks PMA up to 2 years of corrected age. Additional mechanistic elements are investigating underlying mechanisms of azithromycin action by studying the effects of azithromycin on the lung and stool microbiome, tracheal aspirate cytokines and proteome, and on antibiotic resistance against azithromycin in lung and stool samples obtained in the first 3 weeks of life.

## Methods/design

### Main objectives

The main objectives of the AZTEC-FU study are to compare respiratory, neurodevelopmental, growth and mortality outcomes at 1 and 2 years of corrected age in infants who received azithromycin or placebo in the early neonatal period.

**Fig. 1:**
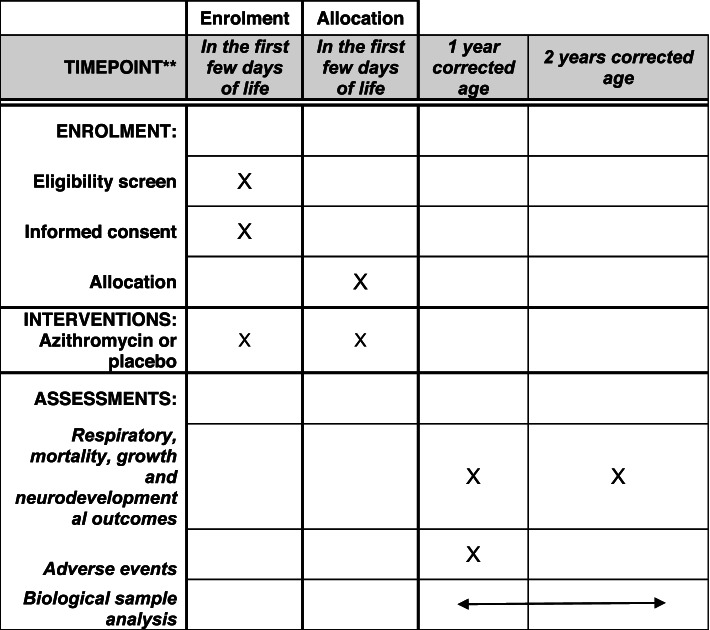
SPIRIT figure

The specific aims are to compare between the treated and placebo groups (Fig. 1):

Mortality:

1. Total number of deaths occurring from birth to 1- and 2-years of corrected age will be reported as will those occurring (a) from birth to 36 weeks’ PMA (which will have been reported in the AZTEC trial), (b) 36 weeks to never discharged and (c) after discharge.

Respiratory:

The main outcome will be parent reported wheezing at 1 year of corrected age. The following outcomes will also be reported:

1. Length of the initial neonatal stay in hospital
2. Number of infants discharged home on domiciliary ambulatory oxygen
3. Length of oxygen supplementation (including at home)
4. Parent reported wheeze from discharge to 2 years of corrected age
5. Number of respiratory hospital admissions up to 1 year and 2 years of corrected age
6. Number of prescribed respiratory drugs
7. Survival (from birth) without parent reported wheeze at 1 year of and 2 years of corrected age

Neurodevelopmental:

The main outcome will be survival (from birth) without combined moderate/severe neurodevelopmental disability at 2 years of corrected age. The following outcomes will also be reported at 2 years of corrected age:

1. Moderate/severe neurodevelopmental disability
2. The proportion of infants who develop moderate-severe neurodevelopmental impairment, defined as Parent Report of Children’s Abilities–Revised (PARCA-R) questionnaire score of < 44
3. Overall motor score as well as fine and gross motors scores
4. Overall language scores as well as expressive and receptive language scores
5. Overall cognition score

Combined moderate/severe neurodevelopmental will be defined, as previously [28, 29] as any of the following:

- Moderate or severe visual impairment (reduced vision uncorrected with aids, blindness in one eye with good vision in the contralateral eye or blindness or light perception only)
- Moderate or severe hearing impairment (hearing loss corrected with aids, some hearing loss uncorrected by aids, or deafness)
- Moderate or severe gross motor impairment (inability to walk or sit independently)
- Moderate or severe cognitive impairment will be defined using PARCA-R or by using clinical data if PARCA-R scores are missing. Total PARCA-R scores of less than 44 (range, 0 to 158, with lower scores indicating greater impairment) will be used to identify children with moderate or severe developmental impairment

Growth:

1. Weight, height and head circumference will be reported at 1 and 2 years of corrected age after adjusting for sex and gestation

Adverse events

1. We shall also report the rates of reported pyloric stenosis in the active treatment and placebo groups at 1 year of corrected age

Biological sample analysis:

1. The respiratory and stool samples collected during the AZTEC trial [27] will additionally be analysed to investigate whether, when compared to the placebo group, azithromycin alters the cytokine, proteomic and microbiome profiles in lung or stool samples as well as any modification that occurs in those colonised by *Ureaplasma* spp

### Design

AZTEC is a double-blind, randomised, placebo-controlled trial using azithromycin or placebo as previously described [27]. The AZTEC-FU study will continue to follow-up the infants recruited to AZTEC up to 2 years of corrected age. The participants and researchers will continue to remain blinded for the AZTEC-FU study.

**Fig. 2:**
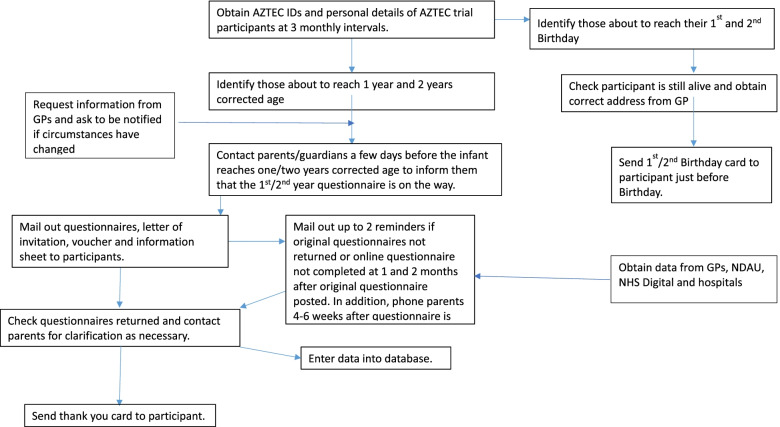
Flow Chart of Process

Data between 36 weeks’ PMA and discharge from the neonatal unit are collected from a dedicated discharge clinical record form (CRF), neonatal discharge summaries and clinic letters, data held in the National Neonatal Research Database (NNRD) and from the parents. When the infants enrolled in the AZTEC trial reach 1-year and 2-years of corrected age, we are contacting the parents/guardians by mailing them validated (a) respiratory and (b) neurodevelopmental questionnaires. In addition, we are asking the parents/guardians for (c) information on any hospital admissions and (d) growth data of their babies from the Red Book system in the UK, which records growth data by Health Visitors, and at local hospital follow-up clinics. An option is available to complete the questionnaire on a secure online site. In parallel, we are contacting local hospitals for clinic letters from which relevant data are extracted: including (a) respiratory symptoms, (b) developmental progress, (c) growth, (d) hospital admissions, and (e) any medication especially respiratory and antibiotic usage. In addition, we are also contacting the General Practitioners for clinic letters, discharge summaries and a copy of the GP record for the children to provide an additional route to collect the above information. Data will also be obtained from NHS Digital for information on hospital admissions and outpatients’ attendances and from the data held in the National Neonatal Research Database (NNRD) at Imperial College, a central repository where most of the UK neonatal units send their clinical data [30] (Fig. 2).

### Patient and public involvement

Input from parents of preterm-born children was given during the grant application on the design and conduct of the study and parents were part of the Trials Management Group and Trials Steering Committee. Parents with previous preterm-born children as well as senior neonatal nurses and doctors also reviewed the information sheets and questionnaires. Amendments were made after their input.

### Setting

Infants have been enrolled on the AZTEC trial from 28 tertiary neonatal units and approximately 77 step-down units in the UK. Where parental consent permits, they are being followed up at 1 and 2 years of corrected age.

### Inclusion criteria

1. Participation in the AZTEC trial
2. Survival at 36 weeks’ PMA
3. Consent provided by the parents/guardians to be contacted for follow-up at 1 and 2 years of corrected age

### Exclusion criteria

1. Withdrawal from AZTEC
2. Parents/guardians did not provide consent to follow-up at 1 and 2 years corrected age
3. Death prior to 36 weeks’ PMA
4. Post-discharge survival not confirmed

### Trial intervention

As previously described [27], infants born at < 30 weeks’ gestation, within 72 h of birth, were treated intravenously for ten days with 20 mg/kg azithromycin for 3 days, followed by a further 7 days of 10 mg/kg or with similarly constituted and administered placebo.

### Trial procedures

#### Site selection and training

Tertiary and step-down units who were involved in AZTEC have been invited to participate. Sites only need to provide copies of clinic letters and discharge summaries; thus, no formal training is required.

#### Participant recruitment

During the AZTEC trial, the AZTEC-FU study is also discussed, and written informed consent is obtained from the parents/guardians for follow-up to 2 years of corrected age.

#### Screening and consent

Eligible infants were screened postnatally against the inclusion and exclusion criteria as previously described [27]. At the initial consent procedure for inclusion in the AZTEC study, the parents/guardians optionally consented to follow-up at 1 and 2 years of corrected age with an additional option to contact the infant’s general practitioner (GP). In addition, the parents/guardians optionally consented for their babies’ data to be reviewed on relevant databases such as NHS Digital and data held in the NNRD. Any participant withdrawals or deaths during the initial neonatal unit stay are communicated by the AZTEC team to the AZTEC-FU study team. Continued consent is also presumed if the parents/guardians complete and return the completed questionnaire at 1 and 2 years of corrected age. The parents/guardians have the right to withdraw from the study at any time without affecting their infant’s clinical care is clearly communicated.

#### Randomisation

The recruited infants were randomised to either active treatment or placebo as previously described [27]. Double blinding is being maintained for the AZTEC-FU study.

#### Data collection

All collected data are securely and confidentially stored with restricted access on Cardiff University servers according to the university’s security policy.

#### Questionnaires at 1- and 2-year’s corrected age

For those who consented to follow-up, a questionnaire including a covering letter explaining the study, an information sheet explaining the questionnaire, a stamped questionnaire return envelope and a voucher to thank the parents for their baby’s participation in the study are mailed. The letter also contains a personalised quick response (QR) code and website URL link which they can use if they wish to complete the questionnaire via a secure online website (Online Surveys, Jisc, Bristol, UK).

The questionnaire at 1-year corrected age captures respiratory symptoms, the infant’s and family history of atopy, number of course of antibiotics, number of hospital admissions, maternal smoking during pregnancy and current smoking in the family who share the house with the infant, information on siblings, as well as general developmental questions and entries for the infant’s weight, length/height and head circumference. The questionnaire also specifically asks if the infant has had pyloric stenosis [21].

At 2 years of corrected age, the questionnaire captures the same information as year one, but with greater detailed neurodevelopment data captured via using the PARCA-R questionnaire which has been validated against Bayley’s Scales of Infant Development Version III assessments [31].

#### Data from general practitioners

At 1 and 2 years of corrected age for the infants whose parents/guardians have consented to the AZTEC-FU study, the GPs are contacted for copies of the infant’s notes including medical or surgical consultations, hospital discharge summaries and outpatient clinic or in-patient letters. Data is being extracted from these records by the AZTEC-FU study team including information on number of hospital admissions, diagnoses, duration of home oxygen therapy (if applicable) and drug history.

Data held in the NNRD which collates neonatal data provided for each neonatal admission by almost all neonatal units in the UK. Data collected includes information on initial neonatal admission as well as neurodevelopmental data at 2 years of corrected age, most commonly assessed by using Bayley’s Scales of Infant Development Version III, but during and after the COVID-19 pandemic many units are increasingly using PARCA-R as a screening tool. Data held in the NNRD is being provided annually for each infant recruited to the AZTEC trial for infant’s whose parents/guardians have provided appropriate consent.

#### NHS digital

It is anticipated that data for infants recruited to the AZTEC trial can be obtained from NHS Digital, once the relevant permissions have been obtained for infants whose parents/guardians have provided appropriate consent for access to the infant’s data from relevant databases. NHS Digital data should provide additional and confirmatory data obtained from the various sources outlined above including hospital and general practitioner records.

#### Hospital data

At 1 and 2 years corrected age, the infant’s local hospital is being contacted if we have the relevant consent from the parents/guardians to supply the infant’s hospital discharge summaries and clinic letters.

The local hospital is being identified from the infant’s address and from information from the recruiting hospital and the step-down hospitals. This information is requested from each local principle investigator when the infants reach 1 and 2 years of corrected age.

### Data analysis

#### Sample size

The initial sample size was calculated for the main AZTEC trial: for 12% improvement of survival without CLD, with an alpha of 0.05 and power of 0.90, would require 796 including 10% drop out. The study recruitment completed in March 2022 recruiting 799 infants. For the current study, the power available will depend on returns of questionnaires and collection of data from a wide variety of sources. Mortality between 36^+1^ weeks and 1 year of corrected age is estimated to be < 5% (with negligible occurring in the second year) and the baseline wheezing rate at 1 year of corrected age for children born ≤ 30 weeks’ gestation is estimated at 60–65% [6] and 50–55% for moderate/severe neurodevelopmental disability at 2 years of corrected age [28, 29]. The tables show the effect size for the parent-reported wheezing at any time during first year of life (Table 1) and for survival without developing moderate/severe neurodevelopmental at 2 years corrected age (Table 2) at an alpha of 0.05 and power of 0.80 and 0.90. It is reasonable to expect approximately 70–75% outcome data from the anticipated 700–725 survivors at 36 weeks’ PMA for the follow-up studies, i.e. approximately 500. These should be able to detect differences of 12.7% at power of 0.80 and alpha of 0.05 for parent reported wheezing at 1-year corrected age between the azithromycin and placebo groups. A similar 12.7% difference should be detectable for in survival without developing moderate/severe neurodisability at 2 years of corrected age between the two treatment arms.

**Table 1:** Estimated effect size for respiratory outcome (wheeze at any time during first year of life) for anticipated questionnaire returns

Size per group	Prevalence of wheezing after treatment	Detectable effect size
**Power 0.8, alpha 0.05, initial incidence of wheeze 65%**		
350	54.3%	10.7%
300	53.5%	11.5%
250	52.3%	12.7%
200	50.7%	14.3%
**Power 0.9, alpha 0.05, initial incidence of wheeze 65%**		
350	52.7%	12.3%
300	51.7%	13.3%
250	50.4%	14.6%
200	48.5%	16.5%

**Table 2:** Estimated effect size for neurodevelopmental outcome (survival at 2 years without moderate/severe neurodevelopmental impairment) for anticipated questionnaire returns

Size per group	Survival without moderate/severe neurodevelopmental impairment	Detectable effect size
**Power 0.8, alpha 0.05, initial incidence of survival without moderate/severe neurodevelopmental impairment of 50%**		
350	60.7%	10.7%
300	61.6%	11.6%
250	62.7%	12.7%
200	64.4%	14.6%
**Power 0.9, alpha 0.05, initial incidence of survival without moderate/severe neurodevelopmental impairment of 50%**		
350	62.3%	12.3%
300	63.3%	12.3%
250	64.6%	14.6%
200	66.4%	16.4%

#### Statistical analysis

A statistical analysis plan will be developed prior to the end of the study. Baseline descriptors will be given. Data analyses will be conducted to identify any differences in the respiratory, neurodevelopmental, and growth outcomes, as well as hospital admissions between the placebo and the treatment group using two-by-two tables and chi-square testing. We shall consider *p* < 0.05 as statistically significant and report 95% confidence intervals. We shall also model the data, as in our previous publications [6], to identify early life factors that may result in long-term respiratory/neurodevelopmental abnormalities including presence/absence of *Ureaplasma* spp. at baseline before intervention commenced in the neonatal period; and if the treatment effect is influenced by early life factors, e.g. gestation, sex, and use of mechanical ventilation. Initially, we shall report the univariable data with a view to including any relevant factors into a model (using linear or logistic regression as appropriate). Missing data will be included in the analysis as missing data.

### Secondary analysis

Presence of *Ureaplasma* spp. at baseline (i.e., before randomisation) will be added as an interaction term for the main analyses of parent reported wheezing at 1-year corrected age and for survival without development of moderate/severe neurodevelopmental disability at 2-year corrected age. Main effects and interactions will be reported, as well as the *p*-value for the interaction term. Subgroup-specific estimates (with accompanying 95% confidence intervals) for each outcome will also be derived from model estimates.

## Discussion

The AZTEC trial’s main outcome is to assess if 10-day treatment with azithromycin, versus placebo improves survival without development of CLD. The AZTEC-FU study will follow-up AZTEC trial participants up 2 years corrected age, assessing mortality and important neonatal morbidities including respiratory, neurodevelopmental and growth outcomes. Important safety data will also be collected, including the incidence of potential consequences of early macrolide use, primarily pyloric stenosis.

The use of azithromycin for the prevention of CLD has been examined previously in smaller populations. A meta-analysis of six studies combining data for 469 infants reported potentially reduced rates CLD following neonatal azithromycin administration [32], but highlighted the need for an adequately powered study. A phase IIb study of 121 infants born at < 29 weeks’ gestation assessed *Ureaplasma*-free survival using an early 3-day course of 20mg/kg intravenous azithromycin. Whilst this treatment course proved effective in eradicating *Ureaplasma*, rates of CLD were not significantly different between the groups [33]. When this cohort was followed up at 2 years corrected age, there were no significant differences reported for respiratory or neurological outcomes between the treated and placebo groups, with comparable rates of cough, wheezing, hospitalisation, respiratory medication use and Bayley-III/neurodevelopment questionnaire scores between the two groups [34]. The longer, 10-day course of azithromycin being studied in AZTEC not only provides an antimicrobial effect against *Ureaplasma* but also provides an effective anti-inflammatory effect against the often reported early pulmonary inflammation [23, 35] that may confer additional benefit for the prevention of CLD. The AZTEC trial is not only sufficiently powered to address this question; it will also provide a larger cohort for follow-up and assessment of longer-term outcomes following early azithromycin use. Infants with CLD are known to be at increased risk of poorer respiratory health in the first 2 years of life, with increased parent-reported wheeze [36] and increased hospital admissions for respiratory diseases [4, 5], with a longer-term risk of decreased lung function [8]. If AZTEC proves effective at reducing rates of CLD for infants born < 30 weeks gestation, it will be important to follow these infants to see if there are longer-term benefits. For the AZTEC-FU study, we are aiming to recruit 500 infants which will enable detection of 12.7% improvement in parent reported wheeze and a similar difference for survival (from birth) without development of moderate/severe neurodevelopmental impairment between the azithromycin and placebo groups. Regardless of the success of the AZTEC trial, it is important to continue to follow-up these infants, as previous follow-up studies examining early neonatal interventions have demonstrated longer-term respiratory benefits, often without showing short-term benefits [37]. For example, despite early elective high frequency oscillatory ventilation not conferring a benefit on reducing rates of death or CLD in the neonatal period, infants treated with this ventilation modality had significantly improved lung function at 11–14 years of age [38].

Neurodevelopmental outcome following preterm birth is a key metric for parents and the neonatal community [39]. Preterm birth is inherently associated with increased risk of poorer neurodevelopment [11, 12], with a strong association, and shared risk factors, between CLD and functional and cognitive outcomes [15]. Human studies have shown that azithromycin is widely distributed in the brain tissue [40], with animal models suggesting that azithromycin may also confer neuroprotective benefits. However, human data for the neuroprotective efficacy of azithromycin is currently lacking. Whilst we postulate improved neurodevelopmental outcomes at 2-year corrected age for those infants treated with azithromycin, we shall also investigate if there are detrimental neurodevelopmental effects from the early use of early azithromycin by using two-tailed comparisons. With our anticipated 500 responses, we have estimated that we should be able to detect 12.7% differences for the main respiratory and neurodevelopmental outcomes at 1 and 2 years of corrected age respectively.

The AZTEC-FU studies are also monitoring survival up to 2 years of age, separately reporting deaths at important timepoints up to 2 years of corrected age. Growth is another important postnatal factor, with preterm infants having poorer inpatient growth velocities [41], and those with CLD were noted to have poorer growth once discharged home [16]. We are collecting anthropometric measurements, including weight, length and head circumference at discharge, and over the first 2 years of life from routine clinic appointments and health visitor assessments. If azithromycin proves effective at reducing rates of CLD, it will be important to assess whether that confers growth benefits following discharge home.

In summary, the AZTEC-FU studies are assessing the impact of a 10-day treatment course of early azithromycin in infants born < 30 weeks’ gestation on their mortality, respiratory morbidity, neurodevelopmental outcome and growth during the first 2 years of life. If azithromycin proves beneficial in improving survival without developing CLD in the neonatal period, the AZTEC-FU study should be sufficiently powered to demonstrate if improvements in respiratory symptoms, neurodevelopment outcome or growth occurs by 2 years of corrected age. Most importantly, the AZTEC-FU studies will ensure safety of the neonatal intervention and provide confidence in establishing azithromycin into routine care of preterm-born infants to improve survival without developing CLD.

## Ethics and dissemination

The current version of the AZTEC-FU Protocol is 7.0, dated 28 June 2022. The Wales Research Ethics Committee 3 (Ref 19/WA/0267) has granted ethics permissions and approved all amendments. The Health Research Authority (HRA) has granted NHS permissions. The study is registered on the NIHR portfolio. Cardiff University is the sponsor. The follow-up studies are an investigator-led project funded by Aspire Pharma Ltd. Results will be disseminated in peer-reviewed journals and via national and international conferences.

## Trial status

Protocol version 7.0 dated 28 June 22. Recruitment began in December 2019 and should complete in September 2025.

## Data Availability

CWC, SJK, WJW, DG, and SK will have access to the final dataset.

## Abbreviations

AZTEC: Azithromycin therapy for chronic lung disease of prematurity
AZTEC-FU: Azithromycin therapy for chronic lung disease of prematurity follow-up studies
CLD: Chronic lung disease of prematurity
PMA: Post menstrual age
COPD: Chronic obstructive pulmonary disease
PARCA-R: Parent Report of Children’s Abilities–Revised
NDAU: National Database Analyses Unit
QR: Quick response
